# MeCP2 inhibits proliferation and migration of breast cancer via suppression of epithelial‐mesenchymal transition

**DOI:** 10.1111/jcmm.15428

**Published:** 2020-06-08

**Authors:** Wei Jiang, Yan‐Ling Liang, Yang Liu, Yu‐Yan Chen, Shu‐Ting Yang, Bi‐Rong Li, Ying‐Xian Yu, Yansi Lyu, Rikang Wang

**Affiliations:** ^1^ Department of Anatomy & Histology School of Basic Medical Sciences Shenzhen University Health Science Centre Shenzhen China; ^2^ Guangdong Provincial People’s Hospital’s Nanhai Hospital Foshan China; ^3^ Department of Dermatology Shenzhen University General Hospital Shenzhen China; ^4^ National Pharmaceutical Engineering Center for Solid Preparation in Chinese Herbal Medicine Jiangxi University of Traditional Chinese Medicine Nanchang China

**Keywords:** breast cancer, epithelial‐mesenchymal transition (EMT), invasion, Methyl‐CpG‐binding protein 2 (MeCP2)

## Abstract

Methyl‐CpG‐binding protein 2 (MeCP2) is an important epigenetic regulator for normal neuronal maturation and brain glial cell function. Additionally, MeCP2 is also involved in a variety of cancers, such as breast, prostate, lung, liver and colorectal. However, whether MeCP2 contributes to the progression of breast cancer remains unknown. In the present study, we investigated the role of MeCP2 in cell proliferation, migration and invasion in vitro. We found that knockdown of MeCP2 inhibited expression of epithelial‐mesenchymal transition (EMT)‐related markers in breast cancer cell lines. In conclusion, our study suggests that MeCP2 inhibits proliferation and invasion through suppression of the EMT pathway in breast cancer.

## 1 INTRODUCTION

Breast cancer is the most common malignant disease in the world and the second leading cause of cancer death in women.^1^, ^2^ Triple‐negative breast cancer (TNBC) is a subtype of breast cancer that accounts for about 10%‐20% of the total number of breast cancer cases.^3^, ^4^ TNBC is characterized as being negative for expression of the oestrogen receptor, progesterone receptor and human epidermal growth factor receptor 2 (HER2).^5^

The epithelial‐mesenchymal transition (EMT) is characterized by the acquisition of mesenchymal properties such as the expression of intermediate filaments, Vimentins, Snail and Slug proteins, and the loss of epithelial properties, including the loss of tight junctions and E‐cadherin proteins.^6^, ^7^ EMT is one of the key processes for cancer metastasis in which epithelial cells assume a mesenchymal cell phenotype, thus enhancing migratory and invasive properties of cancers.^8^ EMT is also considered to be the most important and critical step in the process of chemotherapy resistance, local recurrence and distant metastasis.^9^, ^10^ Many studies have found that activating EMT can promote the proliferation and migration in breast cancer cell, while the proliferation and migration also can be inhibited by suppressing EMT.^11^, ^12^ EMT also is an important biological process that is closely related to the dynamic phenotypic plasticity of cells during embryonic development and the progression of tumours.^13^ Recent research revealed that MeCP2 inhibits the expression of HIF‐1 via promoting its methylation in basal‐like breast cancer cells.^14^ And MeCP2 suppresses the progression of pancreatic cancer.^15^ So we speculate that MeCP2 plays a vital role in breast cancer.

The methyl‐CpG‐binding protein 2 (MECP2) gene, located on the long arm region 2 band 8 of the X chromosome (Xq28), is mutated or dysregulated in many nervous system diseases.^16^ As a methylation binding protein, MeCP2 plays important roles in epigenetics.^17^ In recent years, MeCP2 has been shown to regulate genes in vivo in myelomas, as well as in breast and other tissues.^18^ MECP2 has been found to be related to many kinds of cancers including breast and hepatic.^19^ Many reports indicate that MECP2 is a tumour suppressor gene. An early report suggested that MeCP2 promotes the methylation of BRCA1 promoter region and inhibits the expression of BRCA1 in breast cancer.^20^ What's more, a study revealed that MeCP2 transcription level is higher in oestrogen receptor‐positive (ER+) breast cancer than in oestrogen‐negative(ER−) breast cancer, and the activation of ERα inhibits EMT of breast cancer.^21^, ^22^ MeCP2 also can be considered as a novel regulator of EMT, which knockdown inhibits EMT process of glioma cells.^23^ However, in breast cancer, the relationship between MeCP2, EMT and cell proliferation and migration is rarely reported. So we propose the hypothesis that MeCP2 may inhibit the proliferation and migration in breast cancer cell by suppressing EMT. In our study, we demonstrate that MeCP2 plays a tumour suppressor role in breast cancer cells. The loss of MeCP2 expression is an important feature of TNBC, and MeCP2 can inhibit EMT in breast cancer cell lines.

## 2 MATERIALS AND METHODS

### 2.1 Cell culture and transfection

MDA‐MB‐231, SKBR3, MCF‐7, BT‐549 and T47D cell lines were obtained from the Committee on Type Culture Collection of the Chinese Academy of Science and cultured in DMEM supplemented with 10% foetal calf serum (FCS), 1% penicillin/streptomycin and 1% glutamine. During the culture period, the medium was changed with fresh medium and passaged once every 3 or 4 days. After cells reached confluence, they were harvested and used for subsequent experiments. The overexpression pCMV‐MeCP2 plasmid used in MDA‐MB‐231 cells and the control vector pCMV were purchased from Addgene. For MECP2 knockdown experiments in MCF‐7 cells, shMeCP2‐#1, shMeCP2‐#2 and control shMeCP2‐#NC were synthesized by GeneCopoeia.

Cells were cultured in 6‐well plates at a density of 3 × 10^5^ cells per well in 2 mL medium supplemented with serum. 2 µg DNA and 5 µL P3000 were diluted to 125 µL with serum‐free medium, and 6 µL Lipo3000 was diluted to 125 µL with serum‐free medium for transient transfection. The two solutions were mixed gently, incubated at room temperature for 30 minutes, and then, cells were washed once with 2 mL serum‐free medium. The medium was replaced with fresh medium after 6‐8 hours following transfection. Gene activity of the cell extracts was measured 24‐72 hours after transient transfection, depending on cell type and promoter activity.

### 2.2 RNA purification and quantitative real‐time PCR (qPCR) analysis

Purification of RNA was performed using the Trizol reagent, and contaminated DNA was removed using the TURBO DNA‐free Kit (Ambion Inc). cDNA was synthesized from 1 µg of total RNA using the PrimeScript RT reagent kit (Takara Bio) in a 20 µL reaction mixture. For detection of mRNA expression, the SYBR Select Master Mix (Thermo Fisher) and CFX96 Real‐time PCR Detection System (Bio‐Rad) were used to perform qPCR. Primers were designed online using IDT SciTools. Primers used for qPCR are shown in Table 1. In RCS‐p + rates relative to the control rate, the expression change of a target gene was calculated as fold change = 2−(ΔCT, Tg‐ΔCT, control). The PCR protocol used was as follows: 1 minutes at 95°C (10 seconds at 95°C, 15 seconds at 60°C) ×40 cycles and 4°C thereafter.

**Table 1:** Primers and interference sequences used in this study

Gene	Forward primer	Reverse primer
MeCP2	5′TGACCGGGGACCCATGTAT3′	5′CGTCATCGTCGTACAGGAAGAG3′
E‐cadherin	5′‐AAAGGCCCATTTCCTAAAAACCT‐3′	5′‐TGCGTTCTCTATCCAGAGGCT‐3′
Vimentin	5′‐GACAATGCGTCTCTGGCACGTCT‐3′	5′‐TCCGCCTCCTGCAGGTTCTT‐3′
GAPDH	5′‐TGGACTCCACGACGTACTCAG‐3′	5′‐ACATGTTCCAATATGATTCCA‐3′
Snail	5′‐TTCTCACTGCCATGGAATTCC‐3′	5′‐GCAGAGGACACAGAACCAGAAA‐3′
Slug	5′‐GCCTCCAAAAAGCCAAACTACA‐3′	5′‐GAGGATCTCTGGTTGTGGTATGACA‐3′
shMeCP2	5′‐GCUUAAGCAAAGGAAAUCUTT‐3′	5′‐AGAUUUCCUUUGCUUAAGCTT‐3′

### 2.3 Immunoblotting analysis

For detecting protein expression levels, cells were lysed with ice‐cold RIPA lysis buffer (Beyotime). After centrifugation at 12 000 × *g* for 15 minutes at 4°C, the supernatant was collected and protein was quantified using a bicinchoninic acid kit (Beyotime), after which samples were stored at −80°C. Samples were separated using SDS‐polyacrylamide gel electrophoresis, and the loading amount was about 20‐70 µg protein/lane. After transferring the proteins to a polyvinylidene fluoride (PVDF) membrane, the membrane was blocked with 5% fat‐free milk in TBST (Tris‐buffered saline containing 0.1% Tween 20) for 2 hours at room temperature. Proteins of interest were probed using primary antibodies (shown in Table 2) overnight at 4°C. Then, all membranes were incubated for 2 hours at room temperature with appropriate secondary antibodies. Finally, PVDF membranes were scanned using the Mini Chemiluminescent/Fluorescent Imaging and Analysis System (MiniChemi™500) to detect MeCP2, E‐cadherin, Snail, Slug, Vimentin and GAPDH bands.

**Table 2:** Antibodies used in this study

Antibody	Cat.No.	Company	Concentration.	Species
Anti‐MeCP2	ab50005	Abcam	1:1000	Rabbit
Anti‐E‐Ca	3195	CST	1:1000	Rabbit
Anti‐Vimentin	5741	CST	1:1000	Rabbit
Anti‐GAPDH	TA‐08	Zhongshan, jinqiao	1:3000	Mouse
Slug	9585S	CST	1:1000	Rabbit
Anti‐Snail	3879S	CST	1:1000	Rabbit

### 2.4 Immunohistochemistry (IHC) analysis

From January 2004 to December 2005, our group collected tumour tissue from 98 female breast cancer cases (subtype: Luminal 66, TNBC 32) with a pathological diagnosis of invasive ductal carcinoma treated at the Cancer Hospital of Shantou University Medical College. Tumour tissues from these cases were used for MeCP2 staining. Pathological information on the tumours was obtained from patient medical records. The Shantou University Medical Cancer Hospital Research Ethics Committee approved the use of the pathologically assessed tumour tissues, and our study was in accordance with the Code of Ethics of the World Medical Association (Declaration of Helsinki). The tissue was embedded in high‐temperature melted paraffin, and 5 µm thick sections were taken. After deparaffinizing in xylene, sections were hydrated with different concentrations of alcohol and then boiled for 2 minutes using 0.01 mol/L citrate buffer for antigen retrieval. Endogenous peroxidase activity was blocked using hydrogen peroxide, and non‐specific protein binding sites on tissue sections were blocked using normal goat serum. The MeCP2 primary antibody was added to the sections and incubated overnight at 4°C. At the same time, staining of sections was performed using antibodies without immune IgG as negative controls. DAB visualization was then performed, and slides were counterstained with haematoxylin. The intensity of immunostaining and the percentage of positive cells were evaluated and scored. If the cytoplasm and/or membrane of breast cancer cells were brown, the cells were considered to be MeCP2‐positive. Using 400× magnification, five fields were randomly selected for each slice, and 100 cells were observed for each field. The staining intensity scores were divided into four grades: grade 0, no brown; grade 1, weak brown; grade 2, moderate brown; and grade 3, intense brown. The percentage of positive tumour cells was also divided into five grades: grade 0, negative; grade 1, 1%‐25%; grade 2, 26%‐50%; grade 3, 51%‐75%; and grade 4, ≥76%. The staining intensity was multiplied by the percentage of positive tumour cells to obtain a total score. Sections with high expression were defined as a total score ≥ 6, and those with low expression were defined as a total score < 6.^24^

### 2.5 Wound healing assay

MDA‐MB‐231 or MCF‐7 cells were cultured for 24 hours in serum‐free medium; then, a line was made through the cells to simulate an injury using a 2 mm wide pipette‐tip when cells reached 90% confluency. Subsequently, to remove detached cells, they were washed three times with phosphate‐buffered saline, and then, they were allowed to migrate in serum‐free medium. To measure cell migration, photographs were taken (at a magnification of 400×) after 24 hours of MDA‐MB‐231 cell growth or 48 hours of MCF‐7 cell growth. Five fields were chosen randomly for quantitative measurements within the injured areas.

### 2.6 Transwell assay

Cells were first inoculated without serum for 24 hours in Matrigel‐coated/uncoated chambers (8 µm pore size, BD Bioscience). Inoculation of MCF‐7 or MDA‐MB‐231 cells was performed at a cell density of 5 × 10^4^ or 2 × 10^4^ in the upper chamber containing serum‐free medium. Complete medium was added to the bottom chamber. After 48 hours of cell culture, cells were stained with 0.1% gentian violet. Each experiment was repeated three times. Two observers counted cells in five regions of each well.

### 2.7 Colony formation assay

MCF‐7 cells were seeded in 6‐well plates at a density of 3 × 10^5^ cells/mL. After 24 hours of culture, cells were transfected with 2 µg of shMeCP2‐#1 and #2 and 2 µg of shNC (negative control) in turn, using Lipofectamine 3000. MDA‐MB‐231 cells were cultured in 6‐well plates at a density of 1 × 10^5^ cells/mL. After 24 hours of culture, cells were transfected with 2 µg of pCMV‐MeCP2 in turn. After 48 hours following transfection, cells were seeded in 6‐well plates at a density of 1000 cells/well, and selection was performed using 1 mg/mL G418. After 2 or 3 weeks, cells were stained with gentian violet and the colony number was counted and analysed. All data were obtained from three independent experiments.

### 2.8 Statistical analysis

All experiments were performed in triplicate. Student's *t*‐test was used to evaluate the statistical differences between the experimental and control groups. Data are shown as the mean ± standard error of the mean (SEM).

All the statistical analyses were performed using GraphPad Prism version 7.0. For all analyses, *P* < .05 was considered statistically significant.

## 3 RESULTS

### 3.1 The expression of MeCP2 in luminal is higher than that in clinical breast cancer samples and TNBC cell lines

**Figure 1:**
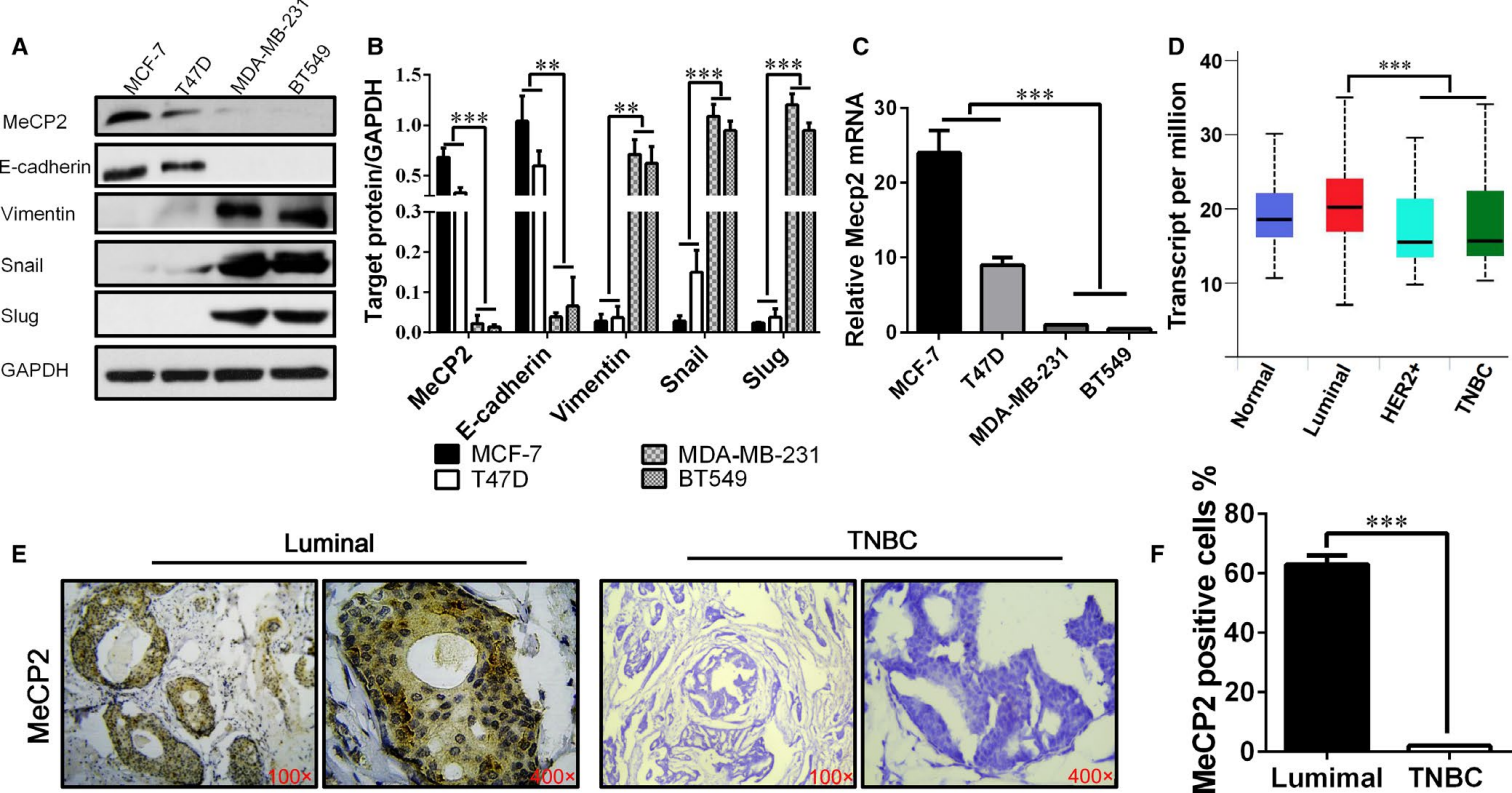
MeCP2 expression is reduced in both TNBC cell lines and human breast cancer samples. A, Western blotting was used to detect the expression of MECP2 and EMT‐related markers such as Snail, Slug, E‐cadherin and Vimentin in breast cancer cell lines MDA‐MB231, BT549, MCF‐7 and T47D. B, Statistical analysis of three experiments for the Western blot band. C, Quantitative analysis of relative MeCP2 mRNA levels in different breast cancer cell lines using real‐time quantitative PCR. D, TCGA data on the expression of MeCP2 in various subtypes of breast cancer. E, MeCP2 staining was performed in representative samples of luminal and triple‐negative breast cancer samples. Magnification, 100×, 400×. F, Statistical analysis of MeCP2 IHC staining. All experiments were performed at least three times and data were statistically analysed by two‐sided *t* test. *P < .05, **P < .01, ***P < .001. Error bars indicate SEM

To investigate the potential role of MeCP2 in breast cancer, we examined the expression of MeCP2 in clinical breast cancer tissues and cell lines. Immunoblotting suggested that MeCP2 was mainly expressed in breast cancer cells of the luminal epithelial subtype, and E‐cadherin expression was increased in T‐47D and MCF‐7 cell lines (Figure 1A and B). The raw data as shown in supplemental Figure S1 and S2 and Table S1 and S2. However, MeCP2 expression was not detected in either basal‐like BT‐549 or MDA‐MB‐231 cells, which express the stromal cell markers Slug, Snail and Vimentin (Figure 1A and B). The raw data as shown in supplemental Figure S1 and S2 and Table S1 and S2. We also measured the expression of MeCP2, E‐cadherin, Snail, Vimentin and Slug mRNA in several breast cancer tissues using real‐time PCR. As expected, high expression of MeCP2 and E‐cadherin mRNA was detected in the ERα‐positive T‐47D and MCF‐7, but not in the basal‐like BT‐549 and MDA‐MB‐231 breast cancer cell lines (Figure 1C). On the contrary, Vimentin, Snail and Slug were relatively highly expressed in both basal‐like breast cancer cell lines (Figure 1C). Breast cancer data in The Cancer Genome Atlas (TCGA) support our results (*P* < .05; Figure 1D). The levels of MeCP2 expression were further analysed in 98 human breast cancer specimens, including 32 triple‐negative and 68 luminal breast cancer cases. IHC results showed that the expression of MeCP2 was higher in luminal than in TNBC samples (Figure 1E and F). Overall, our results suggest that MeCP2 may be associated with the luminal epithelial subtype in breast cancer.

### 3.2 Overexpression of MeCP2 increased the expression of epithelial markers but knockdown of MeCP2 increased the expression of mesenchymal markers

**Figure 2:**
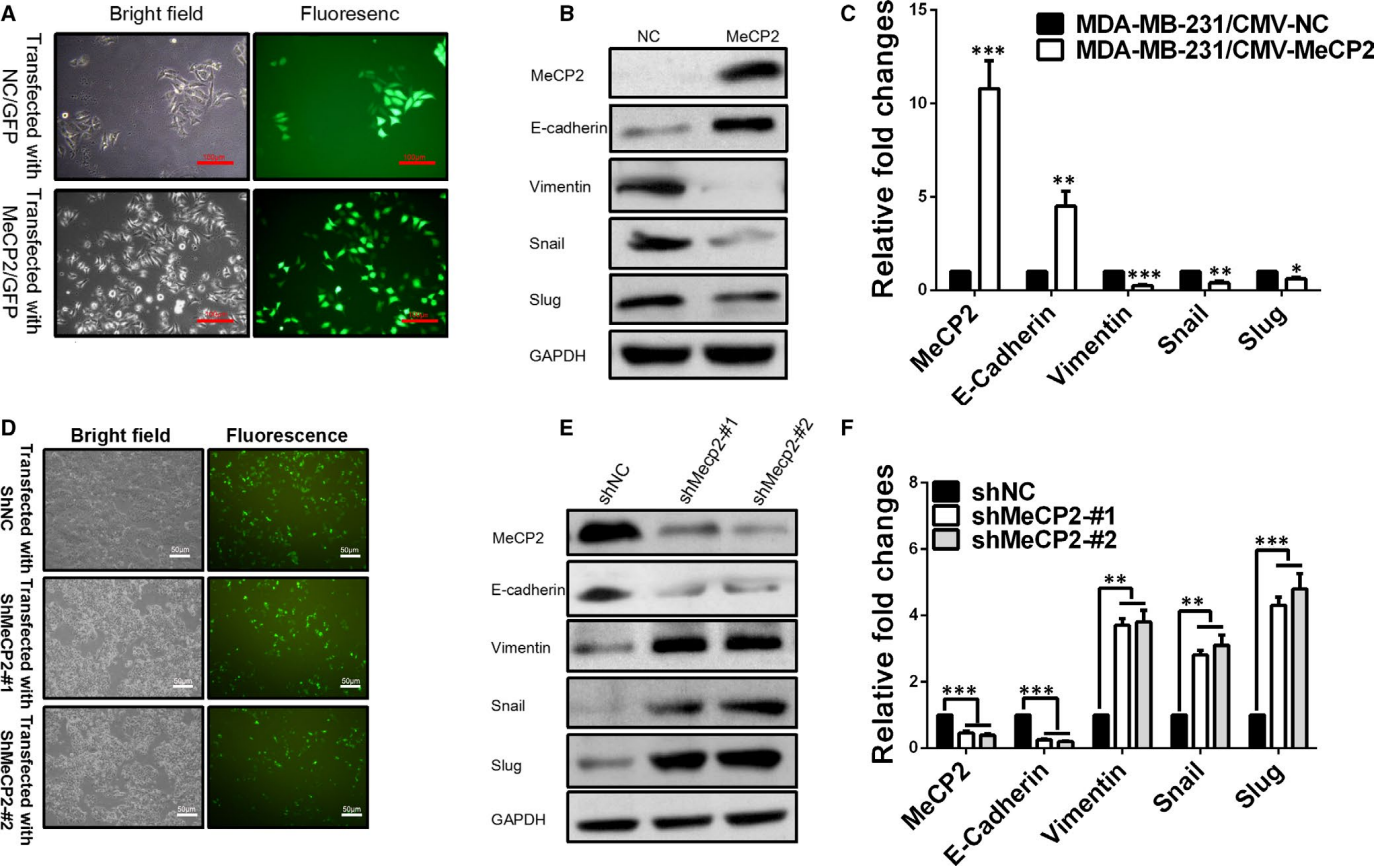
MeCP2 overexpression or knockdown regulates the expression of EMT‐related markers in breast cancer cells. A, Successful transfection of MDA‐MB‐231 cells with an MeCP2 overexpression construct is confirmed by co‐expression of GFP. Scale bars = 100 µm, Magnification, 100×. B(Protein)or C (mRNA) levels for MeCP2 and EMT‐related markers were detected using Western blotting or quantitative real‐time PCR in MDA‐MB‐231 cells that were co‐transfected with the GFP control and MeCP2 vectors. D, ERα‐positive MCF‐7 cells were transiently transfected with shRNAs against MeCP2 (#1 and #2) for 72 h. Scale bars = 50 µm, Magnification, 40×. Western blotting (E) and quantitative real‐time PCR (F)were used to examine the expression of MeCP2 and EMT‐related marker proteins and mRNA. All experiments were performed at least three times, and data were statistically analysed by a two‐sided *t* test. *P < .05, **P < .01, ***P < .001 vs control. Error bars indicate SEM

To investigate the inhibitory effect of MeCP2 on EMT in breast cancer cells, we constructed an MeCP2 expression vector pCMV/MeCP2. Plasmids were transiently transfected into MDA‐MB‐231 cells with low expression of MeCP2. Green fluorescent protein (GFP) was used as a control for transfection, which was considered successful transfection if green fluorescence could be detected (Figure 2A). The results of real‐time PCR and Western blotting showed that MeCP2 and E‐cadherin expression were increased in MDA‐MB‐231 cells transfected with pCMV/MeCP2. mRNA and protein levels were also increased, but Vimentin, Snail and Slug were decreased in these cells (Figure 2B and C). Additionally, we also generated MeCP2 knockdown vectors (shMeCP2‐#1, #2) and the scrambled control vector (shMeCP2‐NC) and then transfected them into MCF‐7 cells (Figure 2A). Successful transfection was confirmed by GFP‐positive cells (Figure 2D). Therefore, real‐time PCR and Western blotting suggested that MeCP2 and E‐cadherin expression in shRNA‐MeCP2/MCF‐7 cells was down‐regulated compared with shRNA‐NC/MCF‐7 cells, but Vimentin, Snail and Slug were up‐regulated (Figure 2E and F). Above results showed that EMT was inhibited by MeCP2 in breast cancer cell lines.

### 3.3 MeCP2 inhibits proliferation, migration and motility of breast cancer cell

**Figure 3:**
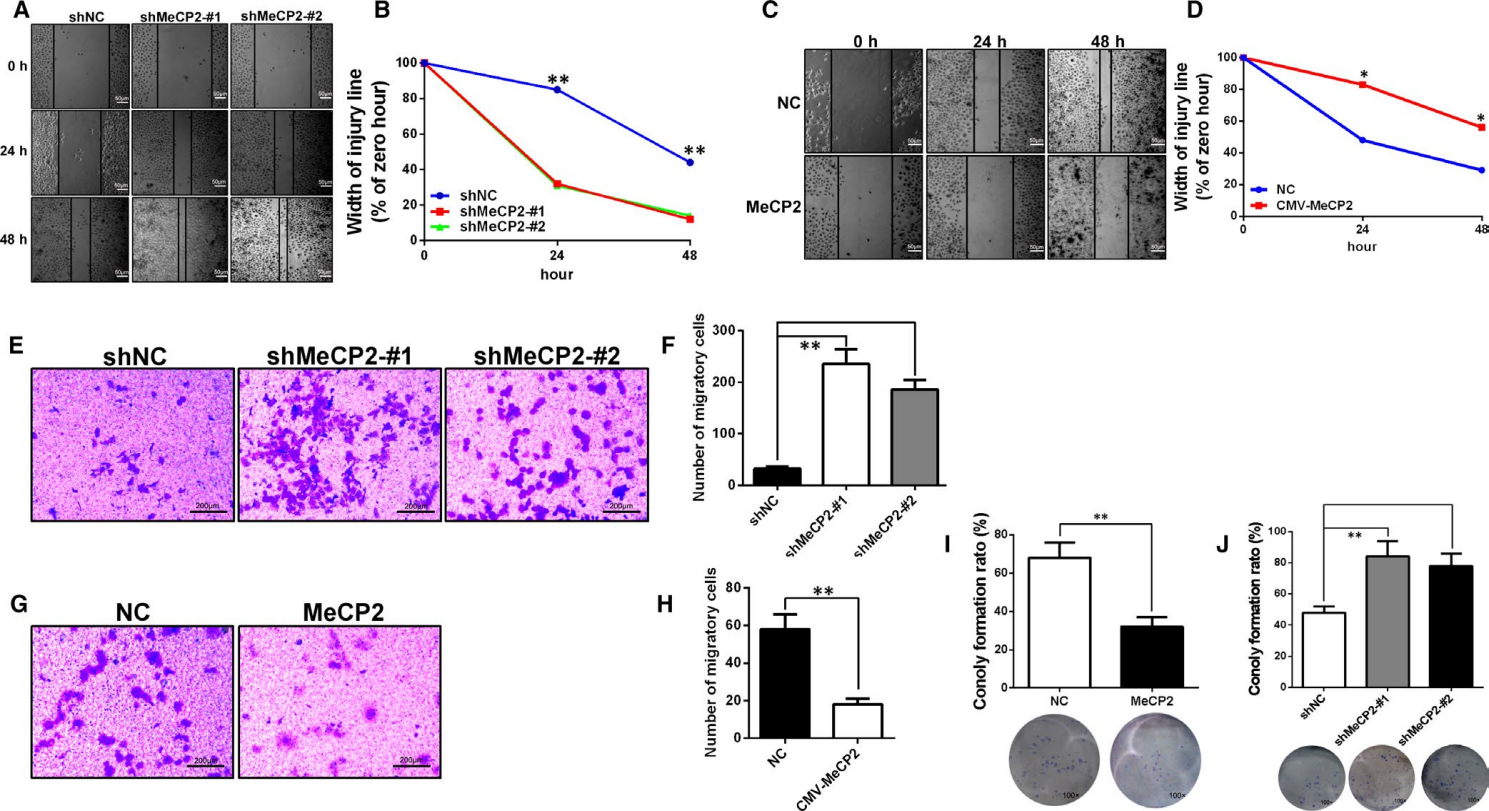
MeCP2 inhibits proliferation, motility and migration in breast cancer cells. A and B, After 48 h of cells culture, the wound healing assay showed that knockdown of MeCP2 in MCF‐7 cells resulted in increased cellular motility. Scale bars = 50 µm, Magnification, 40×. C and D, Overexpression of MeCP2 in MDA‐MB‐231 cells resulted in decreased cellular motility. Scale bars = 50 µm, Magnification, 40×. E and F, Transwell assays in monolayer cultures were performed to assess tumorigenesis of MCF‐7 cells treated with shMeCP2 knockdown vectors. Scale bars = 200 µm, Magnification, 200×. G and H, MDA‐MB‐231 cells transfected with an MeCP2 overexpression vector. Scale bars = 200 µm, Magnification, 200×. I, In the colony formation assay, representative micrographs and quantitative data show the colony formation rate in MDA‐MB‐231 cells that overexpress MeCP2. Magnification, 100×. J, MCF‐7 cells that knocked down for expression of MeCP2. Magnification, 100×. All experiments were performed at least three times, and data were statistically analysed using a Student's *t* test (*P < .05, **P < .01 and ***P < .001 vs control)

The effect of the MeCP2 on the motility and migration of MCF‐7 and MDA‐MB‐231 cells was investigated using wound healing and transwell assays. Wound healing assays demonstrated that MeCP2 knockdown increased cell mobility in MCF‐7 cells (Figure 3A and B), while overexpression of MeCP2 decreased cell mobility in MDA‐MB‐231 cells (Figure 3C and D). In addition, transwell assays showed that MeCP2 silencing caused a 5.8‐fold increase in mobility in MCF‐7 cells compared with the scrambled siRNA (Figure 3E and F). The expression of ectopic MeCP2 was sufficient to decrease invasion (3.2‐fold) of MDA‐MB‐231 cells (Figure 3G and H). To confirm the ability of MeCP2 to affect colony formation, we observed the recovery of colony formation by knocking down MeCP2 in MCF‐7 or overexpressing MeCP2 in MDA‐MB‐231 cells. The results suggested that the colony formation rate was decreased after MeCP2 overexpression in MDA‐MB‐231 cells (Figure 3I), and the colony formation rate increased after knockdown of MeCP2 in MCF‐7 cells (Figure 3J). Thus, these results confirm that MeCP2 inhibited proliferation, motility and migration in breast cancer cell lines.

### 3.4 Longer survival curve for MeCP2 high expression

**Figure 4:**
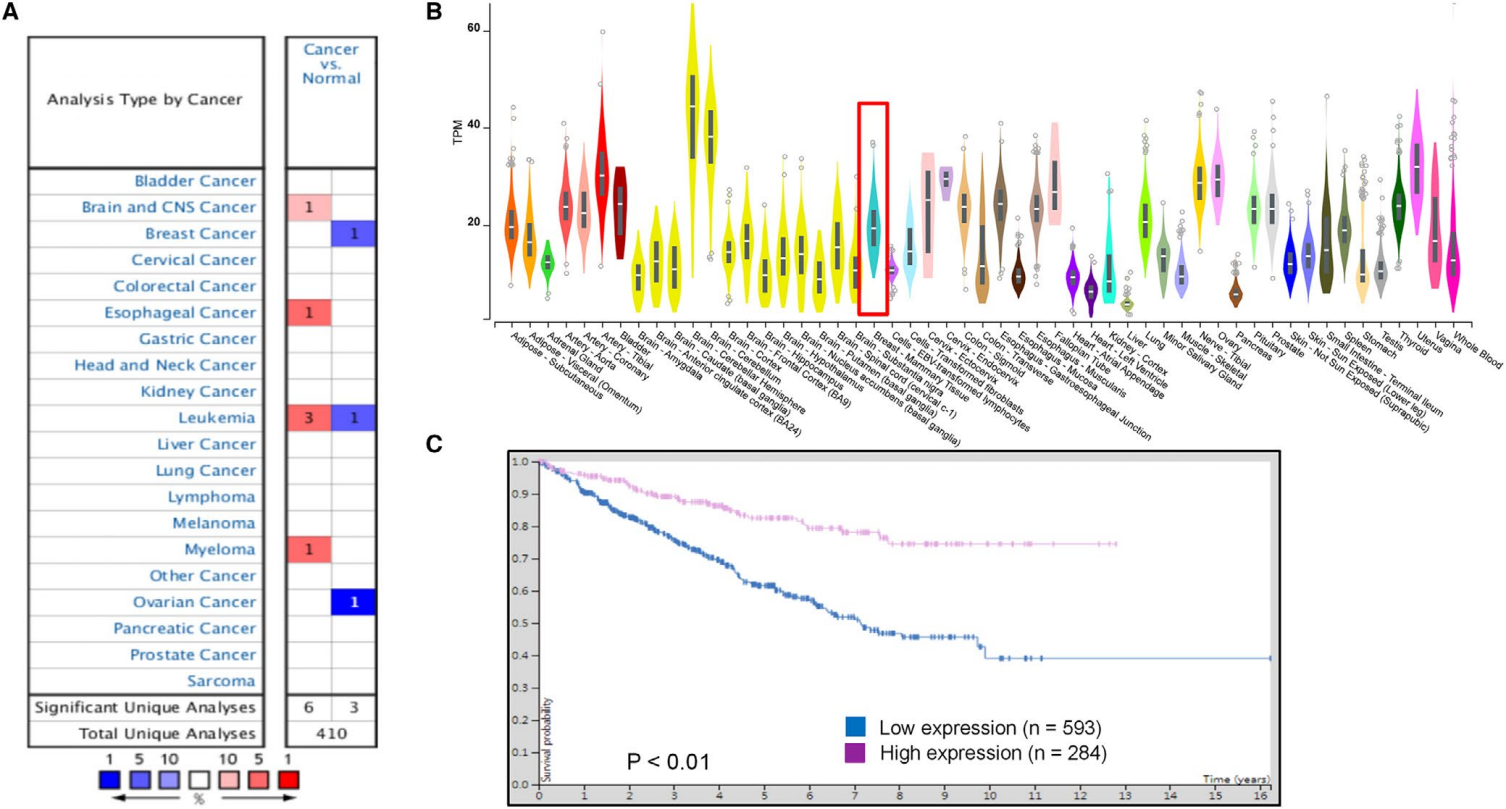
High expression of MeCP2 mRNA was significantly associated with prolongation of overall survival in all breast cancer patients. A, The mRNA expression spectrum of MeCP2 in different cancer types from the ONCOMINE database. B, The graph shows the number of statistically significant datasets for overexpression (Red) or underexpression (Blue) of the target gene mRNA (cancer vs normal tissue). Differences in MeCP2 mRNA expression levels in different tumour tissues from TCGA analysis are shown. C, In all breast cancer patients, high protein levels of MeCP2 were associated with longer OS (data from analysis of the human protein atlas)

ONCOMINE analysis showed that the expression of MeCP2 mRNA in breast cancer was significantly lower than that in normal samples across a wide variety of datasets and in different cancer types (Figure 4A). TCGA database was analysed for the expression of MeCP2 in various cancers, and the results showed that MeCP2 was down‐regulated in breast‐mammary tissue compared with other tissues (Figure 4B). Additionally, we examined data from the human protein atlas and found that highly expressed MeCP2 mRNA was significantly related to the prolongation of overall survival (OS) in all breast cancer patients (Figure 4C). Of note, the results indicated that the high expression of MeCP2 mRNA was significantly associated with prolongation of OS in patients, suggesting that MeCP2 may play a role in breast cancer targeted therapy.

## 4 DISCUSSION

Breast cancer mainly originates from mammary epithelial cells. Of all malignant cancers in women, breast cancer has the highest morbidity and mortality, primarily because of distant metastasis and its resistance to chemoradiotherapy or targeted therapy.^25^, ^26^ For the first time, this study demonstrates that MeCP2 inhibits proliferation and migration via suppressing EMT. So we propose that MeCP2 may act as a potential therapeutic target for breast cancer.

Epigenetic mechanisms such as DNA methylation and histone modification play a key role in the development of cancer.^27^ In many cancers, abnormal methylation of DNA often results in silencing of tumour suppressor genes.^28^ Deletion of DNA methylation occurs in the early stages of tumour development. Both hypomethylation and hypermethylation are indicators of malignant tumours.^29^ The demethylating agent 5‐aza‐2′‐deoxycytidine (5‐aza‐CdR) has been proven to reactivate tumour suppressor genes via suppressing DNA methyltransferase. It is suggested that inhibiting DNA methylation may activate genes expression, especially oncogene and then promote tumour development and metastasis.^30^ However, MeCP2 suppresses LIN28A expression and inhibits cancer development via promoting the methylation of this gene in pancreatic cancer.^15^ In this research, we found that MeCP2 inhibits migration and motility of breast cancer via suppressing EMT.

MeCP2 is an epigenetic regulator that preferentially binds to methylated CpGs and participates in transcriptional inhibition. Additionally, it plays a part in neurodevelopmental disorders. MeCP2 plays a role in haematological malignancies, breast, lung, prostate and liver cancers, as well as other cancers.^31^, ^32^ However, the role of MeCP2 in the progression of breast cancer remains unclear. In our study, we found that E‐cadherin and EMT markers were co‐expressed in a variety of human breast cancer cells, and expression in luminal subtype tumours was higher compared with basal‐like breast cancers, while Vimentin, Snail and Slug were more highly expressed in basal‐like breast cancers. Overexpression of MeCP2 reduced migration and proliferation of breast cancer cells, which was related to the phenotypic conversion of mesenchymal‐to‐epithelial transition (MET). Our results suggest that MeCP2 plays a preventive role in invasive breast cancer.

In this study, we demonstrated for the first time that MeCP2 inhibits proliferation and migration through the EMT pathway in breast cancer. During mammary gland development, MeCP2 expression in mammary luminal‐restricted progenitor/mature luminal cells is higher than that in bi‐potent mammary stem cells, suggesting that MeCP2 maybe play an important role in maintaining luminal breast cancer and inhibiting proliferation of TNBC cell lines. Our study suggests that MeCP2 up‐regulated epithelial markers, such as E‐cadherin, and that MeCP2 knockdown in breast cancer cell lines increased mesenchymal biomarkers, such as Vimentin. Additionally, in the transition from luminal epithelial markers to mesenchymal markers, we found that MeCP2 knockdown significantly promoted the EMT phenotype, including migration and proliferation in vitro. On the contrary, the overexpression of MeCP2 inhibited EMT, migration and invasion of MDA‐MB‐231 cells. These results indicate that MeCP2 can inhibit EMT at least in part. Our results suggest that MeCP2 may be a new biomarker and therapeutic target for breast cancer.

Furthermore, ONCOMINE analysis demonstrated that MeCP2 has lower expression in breast cancer. These data are consistent with the lower expression of MeCP2 in basal‐like breast cancer cell lines that we found. Analysis of TCGA database showed that MeCP2 had lower expression in the breast‐mammary tissue compared with other tissues, which shows a similar pattern with the ONCOMINE database. Finally, survival analysis showed that high expression of MeCP2 was remarkably correlated with better OS in all breast cancer patients.

We therefore hypothesize that MeCP2 may act as an important regulator of several signalling mechanisms associated with EMT. Our results suggest that MeCP2 may inhibit the proliferation, migration and recurrence of breast cancer through suppression of EMT. The EMT signalling pathway contributes to tumour progression characteristics, including invasion, metastasis and angiogenesis.^7^, ^10^, ^33^ Our research demonstrates that MeCP2 expression is significantly correlated with that of EMT signalling pathway‐related markers, indicating that MeCP2 is one of the major factors influencing tumour progression via EMT signalling pathways. Furthermore, clinical outcome data from 98 patients with epithelial breast cancer support our in vitro findings.

In summary, our research suggests that MeCP2 is highly correlated with the progression of breast cancer via suppression of EMT signalling pathways. These results suggest that MeCP2 is a promising prognostic marker and therapeutic target for breast cancer.

## CONFLICT OF INTEREST

The authors declare no conflicts of interest.

## AUTHOR CONTRIBUTIONS

Wei Jiang produced the initial draft of the manuscript and revised it critically for important intellectual content; Yan‐Ling Liang contributed to drafting the manuscript; Rikang Wang conceived and designed the experiments; Yang Liu, Yu‐Yan Chen, Shu‐Ting Yang, Bi‐Rong Li and Ying‐Xian Yu performed the experiments and analysed the data; Yansi Lyu contributed materials and analysis tools. All authors have read and approved the final submitted manuscript.

## Supporting information

Sup info

## Data Availability

All data generated or analysed during this study are included in this published paper.
