# Supplementary material for: MeCP2 inhibits proliferation and migration of breast cancer via suppression of epithelial‐mesenchymal transition
Source: J Cell Mol Med. 2020 Jun 8;24(14):7959–67. doi: 10.1111/jcmm.15428 (PMC7348137; doi:10.1111/jcmm.15428)
Supplement: Supplementary file 1 — Sup info [file JCMM-24-7959-s001.docx]

**Supplementary** **Figures and Tables**


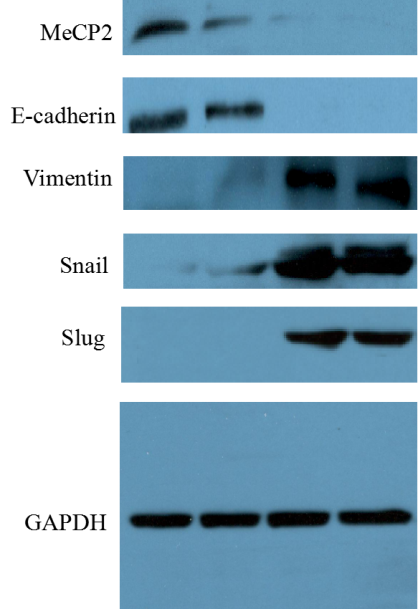

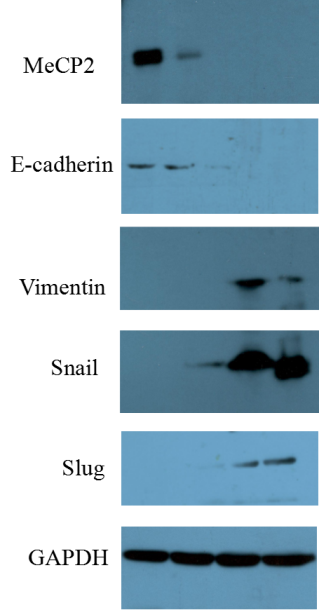

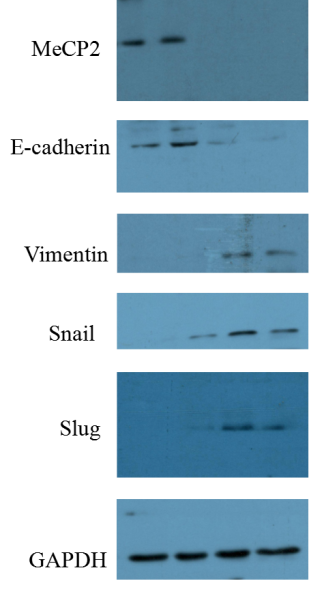


a. The first experiment b. The second experiment c. The third experiment

**Figure S1** **MeCP2 expression is reduced in both TNBC cell lines and human breast cancer samples.** Western-blot band of three experiments (a, b, c).

**Figure S2 MeCP2 expression is reduced in both TNBC cell lines and human breast cancer samples.** The histogram was made by Graphpad software. All experiments were performed at least three times and data were statistically analyzed by two-sided t-test. *P < 0.05, **P<0.01, ***P<0.001. Error bars indicate SEM.


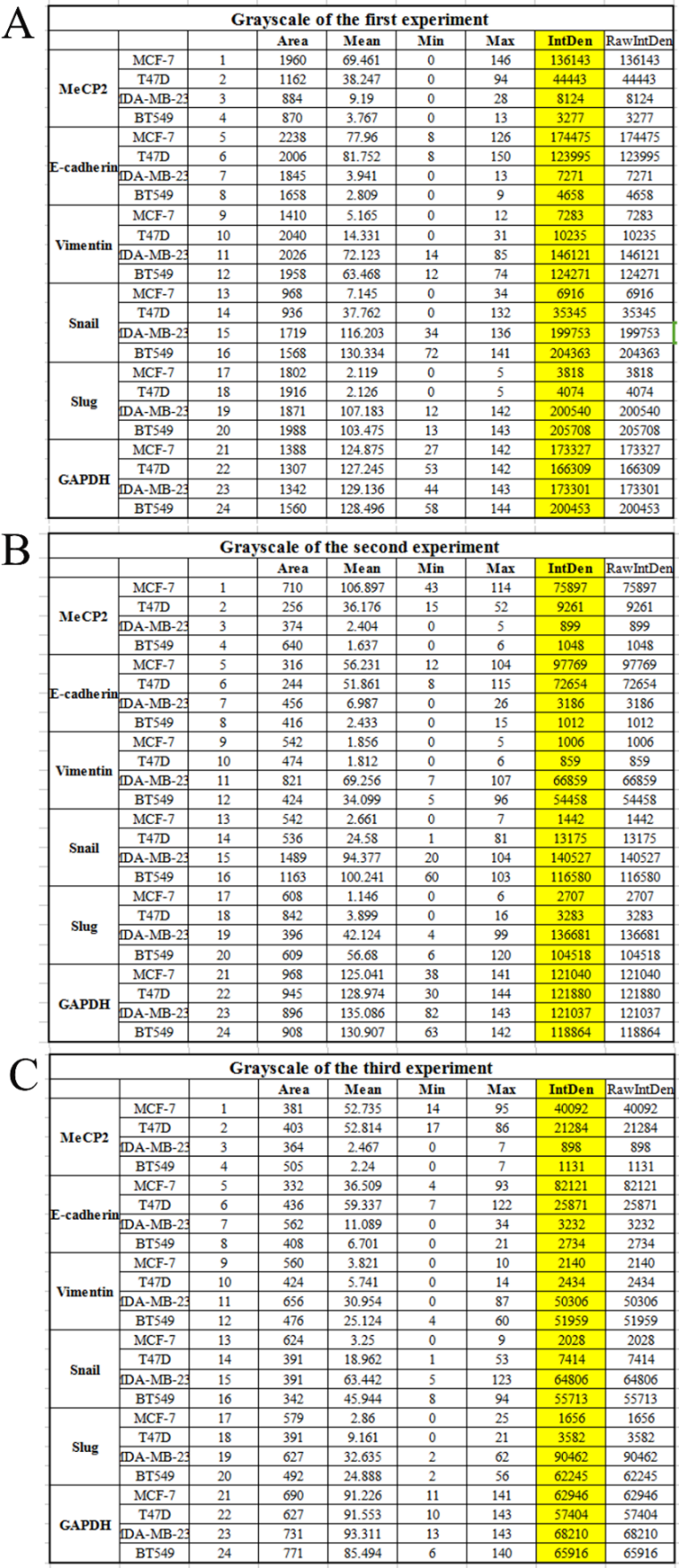


**Table S1** **Grayscale for the western-blot band.** The grayscale measured by Image J v1.8.0（A,B,C）.


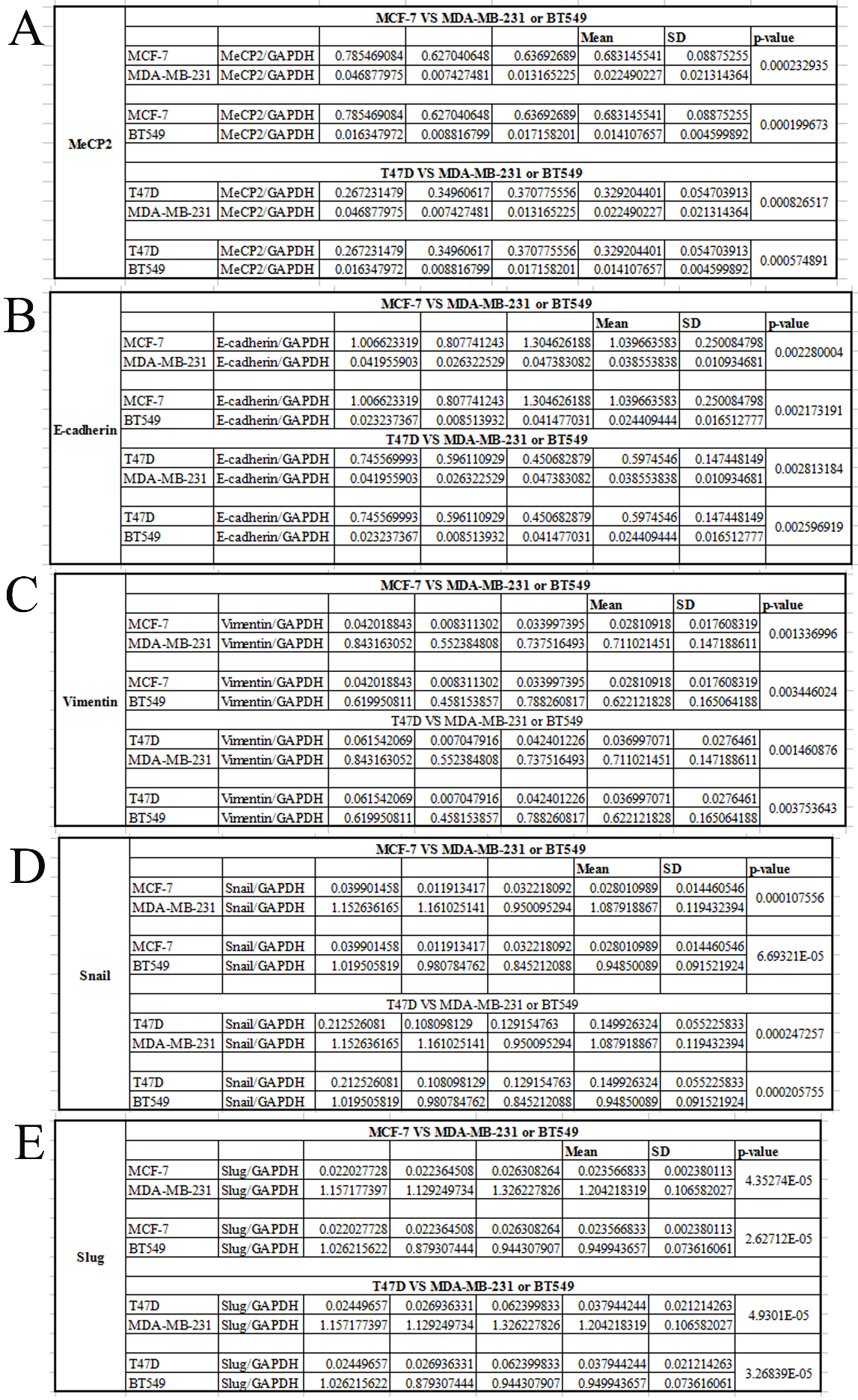


**Table S2 Statistical analysis for the western-blot band using** **Students’ t-test.** The band were analyzed by Students’ t-test (A,B,C,D,E).(P value <0.05 is significant)
